# Work in Great Britain

**Published:** 1930

**Authors:** R. G. Gordon


### Work in Great Britain.

By R. G. GORDON, M.D., D.Sc., F.R.C.P.E.

Mrs. Posthuma has indicated to you the nature of Child Guidance work, and how it is carried on in America. You must realize that there it is essentially a going concern, while here it has scarcely begun. In almost every important city in the States may be found an active Child Guidance Clinic, each with its own problems and its own particular angle of study depending on the leanings of the director and his staff, but all imbued with the enthusiasm of pioneer work which is well worth doing. Work of a similar kind is being done in a less systematic way in other countries, more particularly in Germany, where the "Jugendsamt" takes a real and lively interest in the mental welfare of the children.

Up to the present in this country there has not been a great deal of systematized work done along the lines of child guidance. On the medical side much attention has been paid to the problem of mental deficiency, but with this child guidance has little or nothing to do, except in so far as it may be necessary to recognize the mental defective who comes to the clinic and transfer him to the care of those who are primarily responsible for him. On the social side much has been done in the direction of rescue work, but with this child guidance has still less to do, and must on no account be confused with it. There is a tendency in this country for confusion of mind in both these directions, but it is to be hoped that this will be eradicated, for such misconceptions will do much harm to child guidance: the one by giving to the parents an idea that by bringing their children to the clinic they are confessing that their children are wanting, and the other by bringing into what should be a scientific investigation a moral, religious and even sentimental tone. Psychotherapeutic investigation and treatment of children has been undertaken for several years at the Tavistock Clinic in London, and also at Edinburgh, Portsmouth and Stoke-on-Trent, but this is hardly child guidance, for as Dr. Posthuma has told you, child guidance is more than this, and indeed does not necessarily involve psychotherapeutic measures at all. For the last two years or so the Jewish community has maintained a clinic in the East End of London, under the direction of Drs. Emanuel Miller and Noel Burke, which is on the lines of true child guidance. In the last seven or eight months the demonstration clinic which has been established by the generosity of the American Commonwealth Fund has been in active work in Islington, under the direction of Dr. William Moodie. This is established more or less on the lines of the typical American Child Guidance Clinic as an independent unit, having affinities with other medical services but no close connections with them. Liverpool has just opened a clinic supported by voluntary efforts and by a grant from the Commonwealth Fund. For the last two years or so we have had a Child Guidance Clinic in Bath, run as part of the school medical service.

It may be of interest to consider the possible ways of establishing a clinic and the pros and cons attached to each. It seems to me that there are three possible methods. The clinic may be set up as an independent unit, it may be attached to a mental hospital or a general hospital, and it may be established as part of the preventive health service of the locality. An independent clinic has two obvious advantages and three disadvantages to my mind. The advantages are that it can be entirely dissociated from any idea in the mind of the parent and the child of illness and formal treatment, and the staff are free to develop their work along any particular line which seems to be good to them, a right and proper principle in a new and experimental venture. The chief objection in this country is cost. Separate premises are required, a large separate staff is necessary as Dr. Posthuma has explained, most of whom must be paid, and there seems no obvious means of raising the large sums of money required. Something, no doubt, can and will be done by the Commonwealth Fund to promote the cause of child guidance, but it is neither reasonable nor seemly to expect the American public to finance social services in this country, which, if worth doing, should be supported by ourselves. The only other source of income is through voluntary subscriptions, and with the constant demands on the public purse from taxation on the one hand and the various hospital appeals on the other it is difficult to see where the money is to come from for this somewhat expensive undertaking. Another disadvantage is the reduplication of social services. The independent clinic must have its own social workers, and I do not think the British working-man is too keen on having his home invaded by a number of strangers, however tactful and however well-meaning. If, then, any given home is subject to visits from the health visitors of the school medical department, the social worker of the Child Guidance Clinic and a visitor from one or more church organizations, I for one can hardly wonder if the door is sometimes slammed to and kept slammed. Thirdly, there is the initial difficulty of attracting cases, and to do this the independent clinic must indulge in a certain amount of propaganda. The difficulty about propaganda is that to be effective it has to be somewhat dramatic, and it is easy to make, or at least to imply, extravagant claims, and I think it is most important that child guidance should not do this at the present juncture. We do not really know how much we can do, and indeed how much we are doing, for some figures which I shall give you presently make us pause. To my mind, all that a Child Guidance Clinic should set out to be is a consultative and advisory centre for children with difficulties in the emotional or mental sphere, in exactly the same way as do clinics for the eye, the ear and the heart in their respective spheres. At present these clinics should make no claims to prevent delinquency, psychosis or even psychoneurosis, however much we may hope that the future will show that such claims might have been made and will provide justification for ultimate propaganda along these lines.

The establishment of children's clinics in connection with mental hospitals has the advantage of tapping a source of trained psychiatrists to carry on the work, but it is going to limit the scope of the material to a very large extent, for it is essential that the children should come at the beginning of their difficulties and not when these have reached such proportions as to result in alarming behaviour. With the prejudice against mental illness which is, alas! so rife in this country, it is hardly to be supposed that parents will readily bring their young and only slightly difficult children for advice and treatment to the mental hospital.

The establishment of a clinic in relation to a general

K

hospital has the great advantage that it would have at its command the whole laboratory and other resources of the hospital, and in the case of the teaching hospital opportunities may be given for the training of students to carry on and develop the work. Child guidance work, however, cannot be done in public, and it may be difficult to secure the use of suitable rooms where the clinic can be carried on free from interruptions and disturbances. The atmosphere of hospitals also may not be favourable, since the child may associate it with severe illness, tonsils and adenoid operations and so on. Further, the social services of hospitals in this country are not particularly well developed, and as has already been sufficiently emphasized, this is a very essential part of the work.

To my mind the most likely method is the establishment of the clinic as part of the preventive health services of the community. Child guidance is essentially preventive, and it seems to fit in with the other services provided for this purpose. Through the infant welfare centres and the school medical services there is a close supervision of all the children of the community who are suitable for a clinic of this sort, and it is easy to get children brought to the clinic who give trouble at home or in the school. The parents and children are used to come up to the department for advice, and there is no idea of illness or suffering associated with it. There is an organized system of social work which with a certain amount of adaptation and specialization can be used for the purpose of child guidance with a minimum of overlapping. It is, of course, necessary that the health authority should be sympathetic and helpful, but unless this were so it is obvious that no clinic could exist. Since we run our Clinic in Bath in this way, it may be of interest to give a short description of how we do it.

First, I may say we are fortunate in having the full interest and co-operation of Dr. Blackett, the Medical Officer of Health; secondly, we have the services of Dr. Thomas and Miss Greenall, the School Medical Officer and the Care Secretary, who give a great deal of time and labour entirely voluntarily; and, thirdly, I have been able to enlist the generosity of a lady who has guaranteed the salary of our half-time special social worker, which is practically our only expense.

The children are chiefly drawn from the schools, though the parents are beginning to bring them of their own accord without any prompting from the educational authorities. They have been thoroughly overhauled from the physical side by Dr. Thomas in the course of his school medical inspection. A psychological report, *i.e.* the result of an intelligence test, is provided by him in each case—fortunately he is particularly interested in this work. A report on the home conditions is given by the social worker and a full report is obtained from the school teacher. With all this information available I see the mother, who tells me the story, then the child is brought in. While waiting he has been allowed to play with a collection of toys we have in the waiting-room, and if there is anything significant in his choice or use of the toys this is reported to me. The mother and child are then seen together and, finally, the child by himself. An attempt is made to diagnose the situation. If physical factors are suspected, I refer him to my own out-patient day at the hospital, where I arrange for the necessary investigations. Out of a series of fifty cases the following special conditions of the child have been discovered, the rest being more or less social maladjustments in which the home or the parent was more involved than the child himself:—

| | |
|---|---|
| Psychoneuroses .. .. .. | 3 |
| Glandular defect .. .. .. | 3 |
| Syphilis .. .. .. .. | 2 |
| Chorea .. .. .. .. | 4 |
| Moral defective .. .. .. | 2 |
| Post-encephalitic .. .. .. | 2 |
| Epilepsy .. .. .. .. | 1 |
| | 17 |

Once a month those of us who are specially concerned with the work meet and discuss the cases seen in the interval, hear reports of investigations carried out, and try to determine a course of procedure for the child. On the whole these procedures are quite simple. Once a quarter we have a more formal committee meeting with a solicitor, a parson, a representative of the Education Authority present, so that we may obtain their co-operation in school adjustments, social factors, enlisting the child in Girl Guides and Boy Scouts and so on.

Now to what extent are we useful? To obtain light on the subject we followed up the careers of 100 children who were referred to the School Medical Officer during the last nine years on the supposition that they were mentally defective, but really in the majority of these cases because they were a difficulty in school. Although most of these 100 children were below the 100 per cent. intelligence quotient as estimated by the Terman scale, none of them were certifiable under the Mental Deficiency Act, and continued their careers in the ordinary schools. The full report of the investigation may be found in an article published by us in the *British Medical Journal* of 15th March, 1930. Suffice to say here that although some 50 per cent. showed somewhat serious maladjustments at school age, over 90 per cent. made good later on. Many of these did receive a certain amount of the sort of help that child guidance gives, but of course without any formality of an established clinic, since they were all dealt with before the clinic started. If this is so, is a clinic necessary at all ? I think it is for two reasons. Firstly, if we can avoid the continuance even for a time of these early maladjustments we can save the child a great deal of unhappiness, and although in the majority of cases a reasonably good adjustment seemed to have been made in late adolescence and early adult life, the study of psychoneuroses shows us that early difficulties may later come home to roost, so to speak, and our investigations have in no case gone beyond the twenty-second year. Secondly, from the point of view of research it is important to discover what sort of emotional stresses the average child is put to and the extent to which these can be removed in the early stages. To my mind the essential part of child guidance is the follow-up work, and in our clinic we make no attempt to cover an extensive field, but we do hope to achieve an intensive follow-up of our cases.

I would suggest, then, that in this country it will be necessary to start these clinics as we have always started everything, on a voluntary basis, and only if and when they have really proved their usefulness can we ask the taxpayer to shoulder the burden of full remuneration of the whole of the staff. Perhaps the most important individual is what the Americans call the psychiatrist, though I am not sure that the name is a good one, but he must have a fair amount of experience in psychiatry and psychological medicine, especially in relation to children. He must also be able to keep an eye very widely open for physical defects, such as epilepsy, encephalitis, syphilis and perhaps rheumatic infection, and, above all, he must not be wedded to any particular fad of theory or practice. Similarly, the rest of the staff must avoid the danger which seems to me to be a very real one in America. I refer to the extreme specialism which seems to abolish all sense of proportion and make the individual feel that her methods matter more than anything else. They must all have some knowledge of life, and realize that slight eccentricities may be quite compatible with normality and do not necessarily represent some gross pathological condition.

Child guidance is largely common sense. The psychologist and the social worker must be trained to observe and report facts as they are, and not indulge in their own particular theories and bend the facts to fit them, and, finally, there must be a general enthusiasm and will to co-operation which should not be difficult to bring to bear on this fascinating study.

